# A GP drop-in clinic model providing holistic community care in family hubs: Service evaluation findings and next steps

**DOI:** 10.1016/j.puhip.2026.100759

**Published:** 2026-03-06

**Authors:** Alexandra Macnamara, Joseph Witney, Helen Christmas, Syeda Nudrat, Kate Morton, Sarah Blower

**Affiliations:** a Leeds Teaching Hospitals NHS Foundation Trust, United Kingdom; b Haxby Group, Hull, United Kingdom; c Hull City Council, United Kingdom; d Humber Teaching NHS Foundation Trust, United Kingdom; e University of York, United Kingdom

**Keywords:** Public health, Child health, Health inequalities, Primary care

## Abstract

**Objectives:**

The first few years of life are vital in building the foundations for a child's healthy development, with the needs of the whole family having an impact on how children grow and develop. In addition, many families living in deprived areas face multiple barriers and inequalities in accessing high quality primary care.

A new approach to improving access to primary care was developed in the form of a drop-in GP clinic based in a Family Hub. The model allows families with young children to see a GP as a whole family, with no appointment time restrictions, and the ability to see the same GP at every contact. This provides continuity of care and the opportunity to deliver preventative and holistic care in the context of the whole family.

This service evaluation aimed to explore the reach and impact of the new service on families and practitioners.

**Study design:**

This was a service evaluation based on clinical data and qualitative interviews.

**Methods:**

Clinical data from consultations were used to inform the evaluation, and interviews were undertaken with eight parents and nine Family Hub staff members. Codebook thematic analysis was used to summarise the findings.

**Results:**

In the first six months, 78 family members used the drop-in service in 135 consultations. Most consultations focused on chronic issues such as gastrointestinal issues, mental health and social and neurodiversity concerns.

Four main themes identified from the interview data were: clinical benefits, experiences of accessing and engaging with healthcare, logistics and practicalities, and wider system questions. Families and staff spoke positively about the benefits of the service, including helping to overcome barriers to accessing care, reducing use of emergency services, families having trust and reassurance in the healthcare system, reducing overtreatment, the partnership working with other professionals and families feeling their concerns were taken seriously. Possible limitations included reliance on a single GP, lack of understanding from some families about how to access the service and recognition that the service provided care for pre-school aged children only. Those interviewed felt an expansion of the service into other geographical areas would be beneficial.

**Conclusions:**

The interviews demonstrated a range of benefits from the drop-in model and suggested that this helped to overcome some of the challenges and barriers some families face in accessing usual care in a primary care setting, in addition to providing a more satisfying care experience for both families and clinician.

The next steps for this work will aim to test the viability and cost-effectiveness of this model on a larger scale, including providing the service in another Family Hub and exploring opportunities to test this model in different settings.

## 1 Introduction

### 1.1 Background and rationale

The first years of life are not only a critical time for cognitive, emotional and physical development, but also represent a time when babies are most vulnerable; difficulties experienced in early development can be associated with subsequent poor mental health and physical health outcomes [1,2]. Vulnerability in children is also intrinsically connected to the needs and wellbeing of their caregivers [2,3].

GPs are trained to approach patients' problems holistically and consider children's health in the context of the wider family, however the current 10-minute ‘one patient, one problem’ model does not enable this approach [4]. GPs report not having enough time with each patient, increasing the risk of missed diagnoses early in life, and contributing to low job satisfaction and decisions to leave the role [5].

Alongside these issues, primary care access remains a problem, and many patients face challenges in booking or getting timely appointments [6,7]. Inequalities in capacity to make and attend appointments further exacerbate the issue, particularly for vulnerable families [8].

Hull has the lowest number of GPs per head in the country, meaning that improving access is particularly important, and despite the benefits of seeing the same GP over time, only about a third of Hull patients reported seeing their preferred GP “a lot” or “all” of the time [6].

### 1.2 GP drop-in model

Evidence from the Sure Start programme demonstrated that ‘area-based holistic support’ for families led to improved outcomes [8]. Furthermore, emerging evidence supports co-located models of primary care [9]. Recognising the evidence for integrated and co-located services, alongside local challenges in primary care, a drop-in model based in a Family Hub was proposed and funded as a two-year pilot. This builds on the existing evidence base by providing a different, holistic approach to primary care, co-located within family services.

The pilot consisted of a GP drop-in clinic; providing two sessions per week at a Family Hub in Hull from April 2023, with the aim of delivering accessible place-based care in the community which was preventative, health-orientated and holistic, and met the needs of the family as a whole.

Consultations were in-person, with caregivers seen together with their children. Families saw the same GP every week, and consultations were limited by family need rather than scheduled time. This helped to facilitate a holistic approach to care through which multiple, often interconnected and intergenerational, issues could be addressed.

A logic model was developed to outline how the drop-in clinic might improve outcomes (supplementary information), and a service evaluation was conducted, aiming to explore the following objectives.

○ Reach and usage of the drop-in clinic
○ Parents' and staff experiences of the drop-in clinic
○ Gain insight into how the drop-in clinic might change outcomes

## 2 Methods

### 2.1 Demographic and clinical data were collated from medical records, with patients’ consent

For the interviews, parents were purposively sampled from those attending play sessions. Staff gave permission for their contact details to be shared with the researcher, who invited them to participate. Topic guides were developed iteratively with input from all team members. Semi-structured interviews with eight parents and nine staff members were conducted by an NHS graduate management trainee with previous research experience.

Participants provided informed consent, and interviews were recorded and transcribed, following which, transcripts were analysed using codebook thematic analysis, with an inductive, iterative approach [10]. SN and KM created codes based on the first parent interview. SN then coded the remaining parent interviews. SN and KM reviewed the codes and develop themes. SN then independently coded staff interviews.

## 3 Results

### 3.1 Families using the service

In the first six months, 78 family members used the service in 135 consultations. The eight parents interviewed were aged between 31 and 50 and represented a range of ethnic backgrounds.

### 3.2 Clinical data

Families presented with a range of issues, including infections, dermatological concerns, gastrointestinal issues, mental health problems and social and neurodiversity concerns.

Social and neurodiversity concerns, and early years mental health, featured more commonly than in typical general practice consultations. Concerns rarely required emergency admission.

The model provided families with greater continuity of care, with most families engaging with the service more than once, as well as proactive care, with opportunities for the GP to signpost to community schemes such as the Healthy Start scheme.

### 3.3 Themes

**Fig. 1:**
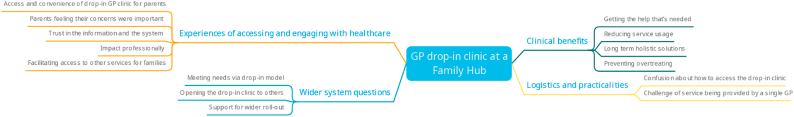
Themes from interview data.

From the interview data, four main themes were identified: clinical benefits, experiences of accessing and engaging with healthcare, logistics and practicalities, and wider system questions (Fig. 1).

### 3.4 Clinical benefits

Parents described how the drop-in clinic helped overcome barriers in accessing primary care. There was also a perception amongst parents that the drop-in clinic helped reduce urgent GP appointment and Emergency Department use.

The drop-in clinic provided opportunity to offer longer term, sustainable holistic solutions, working with families to understand underlying causes and address broader factors, including non-clinical support. The reassurance of continuity also allowed the GP to monitor conditions and reduce the risk of overtreatment.

### 3.5 Experiences of accessing and engaging with healthcare

Parents described the reassurance of being able to see a GP when they needed to, despite clinic availability being limited to two parent-baby groups per week. The convenience of having the drop-in clinic at the Family Hub, which they were already attending, was also noted.

The lack of time constraints for the consultation enabled the GP to provide a holistic, caring approach which helped parents feel validated and confident in raising issues of importance to them. Parents described how this experience was reinforced by having continuity with same GP over time, through the development of both individualised clinical understanding and patient trust. This contrasted with some experiences of usual care where some felt their concerns were not taken seriously due to short appointments with different clinicians.

The importance of unrestricted timing and the GP's approach encouraging parents to talk openly was also noted by staff at the Family Hub, with a suggestion from the GP that the location of the clinic helped families to feel more comfortable discussing their concerns.

Parents described a general sense of trust and belief in the advice they received. For one parent, seeing the GP in person increased their trust in the information they received, and contrasted with their uncertainty regarding telephone appointments in usual care. Seeing the same GP over time was also perceived to enable better quality care, as parents felt better understood. One parent also talked about increased trust in the wider healthcare system as a result.

***“We kind of feel that NHS is looking after us in a good way now. That we've been heard now. We've been looked after. We've been seen. And cared for. That's what NHS is for, isn’t it?”***

Having the GP located at the hub also facilitated partnership working with other professionals. The benefits included awareness of and referrals to appropriate services, clininical decision making with other professionals, and professional relationship building.

### 3.6 Logistics and practicalities

Some parents had not realised they could access the service without registering, suggesting additional information would be helpful.

Having one GP deliver the drop-in clinics meant that the clinic could not be delivered if they were unwell or on leave.

### 3.7 Wider system questions

The interviews demonstrated a differential use of this service compared to usual primary care, and a request for expansion. Some parents described bringing queries they would not take to their usual GP, providing reassurance and potentially supporting early identification and prevention of health issues.

Some families also wanted their older children to have access to the service. Staff suggested expanding the times of the drop-in clinic or allowing more families to attend. Access to the drop-in clinic however, was limited to families with children below five attending the play sessions, to enable the GP to meet the demand without compromising the service aims.

Both parents and staff consistently felt that the service needed to continue and to be provided at other Family Hubs.

## 4 Discussion

The findings demonstrate significant benefits, with emerging evidence of the service meeting the outcomes it was designed to achieve. This pilot was deliberately kept to a small, ring-fenced offer to pilot this model whilst ensuring it didn't promise more than could be delivered; if the service was open to a larger group of people, this may impact on quality and ability to meet need.

There are also risks with having a small service reliant on a single GP, commissioned separately from existing local primary care structures. The benefits experienced by the families and staff gives us a strong responsibility to explore the sustainability of this model, and the potential to expand beyond young families. The next steps need to consider the viability, ethics, and cost-effectiveness of this model on a larger scale.

Limitations of the evaluation included a lack of comparator or economic analysis; a comprehensive evaluation is being planned, which will further explore effectiveness in the current policy context. Recommendations from the evaluation include continuation of the service, providing a mirror service in another Family Hub, further research to understand the impact and exploration of opportunities to use this model in different settings.

## Ethics statement

As a service evaluation, ethical approval was not required to undertake this piece of work. The information sheet explained that participant responses would be kept confidential unless the interviewer was concerned that they or someone else was likely to be harmed. The decision was made not to provide reimbursement for participation due to potential issues if there wasn't the opportunity for everyone to be interviewed.

## Funding

The funding for the drop-in GP clinic was obtained from the Family Hubs and Start for Life grant and Humber and North Yorkshire Integrated Care Board’s health inequalities funding for a two-year pilot. SB and KM time to provide advice and support to the evaluation was provided by the YHARC. SB and KM were supported by the NIHR Yorkshire and Humber Applied Research Collaboration (ARC-YH; Ref:NIHR200166, see https://www.arc-yh.nihr.ac.uk). The views expressed are those of the authors and not necessarily those of the NHS, the NIHR or the Department of Health and Social Care.

## Declaration of competing interest

The authors declare the following financial interests/personal relationships which may be considered as potential competing interests: No specific conflict of interest declared, however please find funding statement below in relation to the research: Funding StatementThe funding for the drop-in GP clinic was obtained from the Family Hubs and Start for Life grant and Humber and North Yorkshire Integrated Care Board's health inequalities funding for a two-year pilot. SB and KM time to provide advice and support to the evaluation was provided by the YHARC. SB and KM were supported by the NIHR Yorkshire and Humber Applied Research Collaboration (ARC-YH; Ref:NIHR200166, see https://www.arc-yh.nihr.ac.uk). The views expressed are those of the authors and not necessarily those of the NHS, the NIHR or the Department of Health and Social Care.
