# Supplementary material for: A GP drop-in clinic model providing holistic community care in family hubs: Service evaluation findings and next steps
Source: Public Health Pract (Oxf). 2026 Mar 6;11:100759. doi: 10.1016/j.puhip.2026.100759 (PMC13010100; doi:10.1016/j.puhip.2026.100759)
Supplement: Multimedia component 1 [file mmc1.docx]

Supplementary information: Logic model


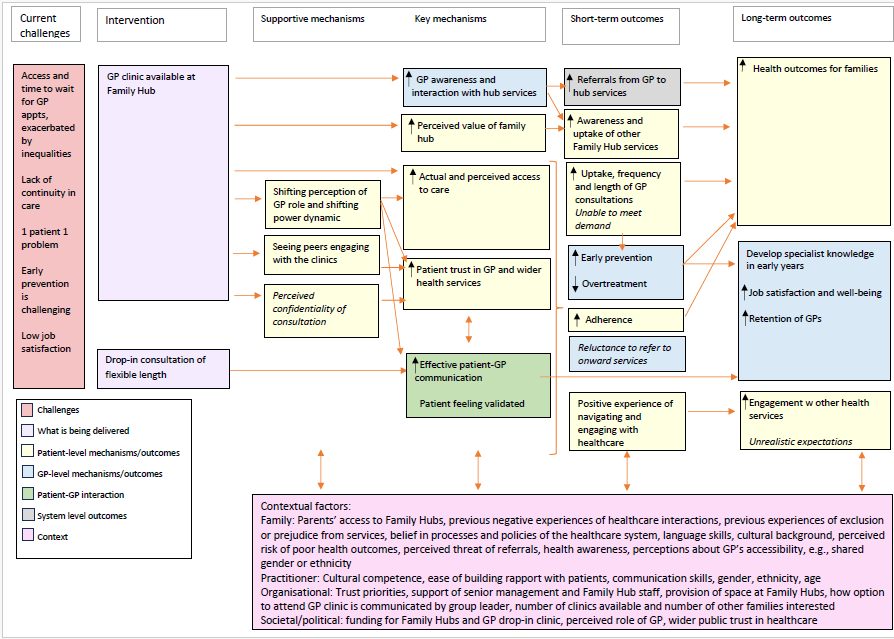


**Supportive mechanisms**

Shifting perception of GP role (supportive mechanism)

The traditional model of 1 patient-1 problem and the strong narrative in society about long waiting times for a GP appointment can promote a feeling of helplessness, such that parents only discuss issues with the GP which they perceive to be serious, ‘worthy’ or clearly defined in their own mind. The location of the GP clinic at the Family Hub, the endorsement of the clinic by the family group leader, and the focus on family health may help shift parents’ perceptions about the role of the GP and what is appropriate to discuss with them, reducing guilt or perceived pressure about using GP’s time for a quick check about issues perceived to be minor, or broader issues around e.g. a child’s behaviour. This shifting perception about the GP could also help underserved groups feel more confident that the GP is there for them and will listen to them.

Shifting power dynamic (supportive mechanism)

Family Hubs are an inclusive space designed for families, and many families may already be quite familiar with their hub through attending community groups. The fact that the GP has come to the hub rather than them having to go to the GP’s own setting may help families feel more confident to speak to the GP, as they are in their comfort zone. Informal feedback from parents at the hub suggests this also applies to children, who are more comfortable at the Family Hub setting than the GP Practice where they may be scared to attend.

Seeing peers engaging with the clinics

Families who attend groups together at the hub may talk to one another and share experiences of using the clinic or may notice that other families similar to them are using the service.

Perceived confidentiality of consultation

To what extent a patient trusts that their information will be kept confidential and not shared with other services.

**Key Mechanisms**

Increased GP awareness and interaction with hub services

Defined as GP awareness of other services and service providers based at the hub.

Influenced by:

- The intervention setting of the GP drop-in clinic being based at the Family Hub, providing the opportunity for better networking and to build relationships with other service providers, such as PMH support groups.

Increased perceived value of Family Hub

Defined as the how useful a family perceives the Family Hub to be for their needs.

Influenced by:

- The intervention setting of the GP drop-in clinic at the Family Hub, as families who already attend the hub may start to perceive it as more of a one-stop-shop for all their needs because of the provision of a drop-in GP clinic, and other local families who had not engaged in the hub previously might become aware of the drop-in GP clinic and start to attend family groups at the hub in order to access it.

Increased actual and perceived access to care

Defined as how easily the parent can actually access the GP, and how easily they perceive they can access a GP, including to what extent it is appropriate and acceptable to do so. It has been defined as “the ability to receive the appropriate care from a proper healthcare provider, at the right time and place, depending on the context” (Saurman, 2016)

Influenced by:

- *GP clinic available at Family Hub (intervention)*
  The physical setup of the clinic at a regular time slot, in a location where families would be anyway, could make it easier for families to attend the clinic. The lack of appointments can make it feel less formal and more opportunistic to consult the GP, and removes barriers around speaking to a receptionist at a GP Surgery and having to plan a time to come in.
- *Shifting perception of GP role (supportive mechanism)*
  Through a changing perception of what the GP is there for, patients perceive better access to the GP as they realise they can approach them for minor concerns or broader queries.
- *Shifting power dynamic (supportive mechanism)*Families are theorised to feel that the GP is more accessible when the GP has come into their space rather than them having to visit the GP Practice.

Increased patient trust in GP and wider health services

Patient trust in GP has been defined as “a set of expectations that the healthcare provider will do the best for the patient, and with good will, recognising the patient’s vulnerability” (Rasiah et al., 2020).

It’s about believing that the GP will act in their best interests (Hall et al., 2001). This includes believing that the GP will treat you fairly, and not feeling judged.

Influenced by:

- *Shifting perception of GP role (supportive mechanism)*The belief that the GP is genuinely interested in even minor or poorly defined concerns and will take a holistic approach to their family is theorised to increase trust, as it suggests the GP will act in their family’s best interests.
- *Shifting power dynamic (supportive mechanism)*

The GP is more visible and is reliably available on a regular basis, which increases trust.

- *Seeing peers engaging with the service (supportive mechanism)*

Seeing other families using the GP drop-in clinic who are perceived as similar to their family may help parents trust that the GP will do their best for them.

- *Perceived confidentiality of the consultation (supportive mechanism)*

The clinic being located in the Family Hub may make some parents uncertain about who will see their consultation notes, especially if a family is concerned about being referred to social services or if they share concerns about their mental health with the GP. Alternatively, this setting within the Family Hub may actually improve trust as families perceive that the GP is there to help them, not as part of the system, and will be more likely to keep their notes confidential.

- *Effective patient-GP communication (another key mechanism)*

A two-way relationship is theorised between patient-GP communication and trust, whereby having effective two-way conversations with the GP increases trust that they care about your outcomes, which in turn improves communication as the patient is willing to share more information.

Effective patient-GP communication

Effective patient-GP communication is defined as “two-way communication (spoken, written and non-verbal) that engages patients in decision making and care planning. It is tailored, open, honest, and respectful and there is an opportunity for clarification and feedback” (Australian Commission on Safety and Quality in Health Care). It includes the patient feeling willing to disclose information, and the GP being able to make holistic care decisions.

Influenced by:

- *Shifting perception of GP role (supportive mechanism)*

Perceiving that the GP is accessible and committed to improving your family’s health outcomes could facilitate more effective communication as the parent is willing to share more information.

- *Shifting power dynamic (supportive mechanism)*

The consultation taking place in a more comfortable and familiar environment may facilitate parents to share more information.

- *Drop-in consultation of flexible length (intervention)*

There are various features of the drop-in consultation that may facilitate effective patient-GP communication, such as being in-person, the consultation being for the whole family and not for one individual, the lack of a tight time pressure on the length of the consultation, and the opportunity to continue attending consultations with the same GP allowing rapport to develop.

- *Patient trust in GP and wider health services (Another key mechanism)*

As described above, a two-way relationship is theorised between patient-GP communication and trust, whereby having effective two-way conversations with the GP increases trust that they care about your outcomes, which in turn improves communication as the patient is willing to share more information.

Patient feeling validated

The patient feels their concerns are important, that they have been listened to, and that they are not being brushed off.

*Drop-in consultation of flexible length (intervention)*A key contributor to the patient feeling validated is the lack of time constraints on the appointment, enabling the GP to explore their problem holistically and provide tailored advice.

**Short-term outcomes**

Increased referrals from GP to hub services

Defined as a higher number of referrals made to other services at Family Hubs

Influenced by:

- *GP awareness and interaction with hub services*

The GP may be more likely to refer to hub services as connections are built with service providers through being based in the same place.

Increased awareness and uptake of other Family Hub services

Defined as more families attend Family Hub services.

Influenced by:

- *GP awareness and interaction with hub services*

Families start to use the hub services more due to referral by the GP.

- *Perceived value of Family Hub*

Families attend the hub more often due the increased value of having the drop-in clinic there, and through attending more often become aware of other services available for them.

Uptake, frequency and length of GP consultations

Defined as more one–off appointments and follow-on appointments per family than in usual care at a GP Practice, and longer duration of consultations.

Demand could become too high over time, with the unintended consequence that the clinic is unable to see all families who attend.

Influenced by:

- *Actual and perceived access to care*

As families have easier access to care via the drop-in clinic at the Family Hub, and perceive easier access to care, they will attend GP consultations more frequently.

- *Patient trust in GP and wider services*

As parents trust in the GP improves, they will be more likely to attend appointments more regularly and to have a longer consultation as their increased trust encourages more in-depth conversations.

- *Effective patient-GP communication*

The parent being more willing to disclose information and the GP being more able to engage in holistic decision making will increase the length of consultations and increase the likelihood that the parent will continue to engage with the GP over time.

Increased early prevention and reduced overtreatment

Earlier and increased identification of physical and mental health issues than usual GP Practice care.

Lower number of prescriptions for ‘just in case’ scenarios.

Influenced by:

- *Actual and perceived access to care*

Knowing that parents are more able to return for future consultations enables GPs to make the decision to hold off treatment rather than treating ‘just in case’.

- *Patient trust in GP and wider services*

Greater trust between the GP and parents enables parents to communicate more openly and to share wider associated health issues in a more holistic setting, facilitating earlier prevention.

- *Effective patient-GP communication*

Two-way communication and holistic decision-making facilitates earlier prevention and reduces overtreatment.

Improved adherence

Parents are more likely to start recommended treatment/interventions and to maintain adherence over time in line with GP recommendations.

Influenced by:

- *Actual and perceived access to care*

More regular appointments could improve adherence as GPs and parents can check-in more regularly to discuss how a treatment or intervention is working, or to answer any questions that have emerged since prescribing.

- *Patient trust in GP and wider services*

Parents are more likely to adhere if they trust the GP’s advice.

- *Effective patient-GP communication*

GPs have more time and are more able to explain the rationale for treatments or interventions and how to effectively adhere, enhancing adherence.

Reluctance to refer to onward services

GP is less likely to refer to onward services or engage in safeguarding procedures than in a usual GP Practice setting.

Influenced by:

- *Actual and perceived access to care*

The GP builds more of a relationship with the parent due to the ongoing consultations over time.

- *Patient trust in GP and wider services*

The increased trust leads the GP to feel more investment in continuing to support the patient.

- *Effective patient-GP communication*

The GP may be concerned that the open communication and trust they have built with the parent would not be continued if they refer the parent on to other services.

Positive experience of navigating and engaging with healthcare

Parents are more satisfied and empowered by their consultation experience.

Influenced by:

- *Actual and perceived access to care*

Easier access makes for a more positive experience, as parents are not having to navigate the process of making an appointment, travelling to the GP Practice etc.

- *Patient trust in GP and wider services*

Feeling trust in the GP leads to a more positive consultation experience, as parents feel listened to and that the GP is genuinely interested in their health.

- *Effective patient-GP communication*

Two-way, open communication helps parents evaluate their consultation more positively as they are able to fully describe the problem and are involved in deciding the next steps.

**Long-term outcomes**

Improved health outcomes for families

Earlier diagnosis, tailored referrals and appropriate prescribing for a wide range of physical and mental health issues for parents and children.

Influenced by:

- *Referrals from GP to hub services*

Referring families to wider support services, such as perinatal mental health support groups or breastfeeding clinics, will help meet their needs and improve wider health outcomes.

- *Awareness and uptake of other Family Hub services*

As parents are either referred to other services or become aware of services themselves by attending the GP clinic at the hub, this engagement with other services will have a positive impact on family health outcomes.

- *Uptake, frequency and length of GP consultations*

The GP will be able to see families who would not normally attend appointments, and see families more regularly over time, both of which will facilitate improved health outcomes by enabling the GP to provide holistic family care.

Longer appointments will have a positive impact on health outcomes as the GP has the time to direct them to specifically relevant services and resources.

- *Early prevention and reduced overtreatment*

Early prevention and reduced overtreatment will improve overall health outcomes for families by preventing problems from escalating and ensuring appropriate diagnosis and treatment.

- *Adherence*

Improved adherence from parents and children to treatments or interventions will improve health outcomes as conditions are optimally managed.

Develop specialist knowledge in early years

Increased GP knowledge about early years healthcare as the GP spends more time with families and hears more details about the holistic family situation and how this impacts on health issues.

*Improved job satisfaction and well-being, retention of GPs*

GP feels more satisfied with their work, has higher mental well-being and fewer GPs leave the NHS.

Influenced by

- *Early prevention and reduced overtreatment*

GPs feeling they have the time and rapport with patients to be able to detect problems early, respond appropriately and reduce overtreatment will help increase their job satisfaction and well-being.

- *Effective patient-GP communication*

GPs gain satisfaction from having effective, meaningful, face-to-face conversations with patients which enable them to do their job better. This will also facilitate the development of specialist knowledge in early years as GPs become more familiar with problems often affecting young families and have increased confidence to support them., enhancing job satisfaction and ultimately retention.

Improved engagement w other health services

Increased uptake and ongoing use of other health services, including referrals. Possible unintended consequence that parents assume that other health services will be able to provide similar drop-in access, and continuity of care. Or parents want to switch GP Practices in order to continue seeing the Family Hub GP.

Influenced by:

- *Positive experience of navigating and engaging with healthcare*

Overall perceptions of healthcare and expectations of positive experiences and outcomes are raised, which leads to more willingness to engage with other health services. However, this could result in disengagement if other care settings are not as quick, responsive, consistent and holistic.

*Contextual factors*

It is theorised that contextual factors will affect all mechanisms, outcomes and relationships between them. This includes factors at the level of the family, practitioner, organisation and society, and more detail is included below:

*Family*:

- Parents’ access to Family Hubs, as those with a Family Hub geographically nearer or with good transport links may be more likely to use the GP clinic
- Previous negative experiences of healthcare interactions, such as long waiting times or lack of positive outcome for addressing the health issue, may reduce the likelihood that parents would be willing to attend a drop-in clinic
- Previous experiences of exclusion or prejudice from services may reduce likelihood of parent engagement if a healthcare practitioner has previously treated them differently because of who they are.
- Holding positive beliefs in the processes and policies of the healthcare system could increase the likelihood of engaging, as these wider beliefs increase the perceived trust and benefit of the intervention.
- Language skills: Being able to speak English may make people more likely to engage in a consultation, due to the lack of a language barrier and confidence in being able to communicate effectively with the GP.
- Cultural background: Some cultures may place higher value on attending the GP for family problems.
- Perceived risk of poor health outcomes: Parents with strong concerns about the possible consequence of health issues may be more likely to attend.
- Perceived threat of referrals: Parents with concerns about being referred to social services or similar may be less likely to attend.
- Health awareness: Parents with higher health literacy and awareness may be more likely to engage.
- Perceptions about GP’s accessibility: Some parents may perceive the GP to be more accessible if they have shared sociodemographic characteristics, such as ethnicity, gender, age group, or other perceived similarities.

*Practitioner:*

- Cultural competence: GP’s sensitivity and understanding of other cultures.
- Ease of building rapport with patients: How easily a GP can build a comfortable relationship with a patient, in which they feel happy to share
- Communication skills: GP’s existing skills for communicating openly and involving the patient in decision-making
- Gender, ethnicity and age: The GP’s gender, ethnicity and age might influence how willing patients are to attend and engage with the drop-in clinic, depending on their own sociodemographic characteristics.

*Organisation:*

- Trust priorities: The extent to which the local trust prioritises drop-in GP clinics compared with competing services
- Support of senior management and Family Hub staff: The amount of support for the GP to have the dedicated clinic time from senior management and Family Hub staff will influence how well this service can be delivered.
- Provision of space at Family Hubs: The GP clinic requires dedicated space at a Family Hub.
- How option to attend GP clinic is communicated by group leader: As families are signposted to the GP clinic by a group leader, the way in which the group leader explains the clinic’s purpose and value could influence engagement.
- Number of clinics available and number of other families interested: How well the clinic works will depend on whether there is enough resource to meet demand.

*Societal/political:*

- Funding for Family Hubs and GP drop-in clinic: The service depends on continued funding for Family Hubs and the GP to have dedicated time to run the drop-in clinic on a regular basis.
- Perceived role of GP: Societal perceptions about the role of the GP may influence the service
- Wider public trust in healthcare: Recent events can influence public trust in healthcare as a whole, which could influence engagement in this service

Saurman, E. (2016). Improving access: modifying Penchansky and Thomas’s Theory of Access. *Journal of Health Services Research & Policy*, *21*(1), 36-39. https://doi.org/10.1177/1355819615600001
